# Associations of visual functions with attitudes about motor vehicle dashboards among older drivers

**DOI:** 10.3389/fnrgo.2022.918781

**Published:** 2022-08-12

**Authors:** Thomas A. Swain, Scott W. Snyder, Jr Gerald McGwin, Cynthia Owsley

**Affiliations:** ^1^Department of Ophthalmology and Visual Sciences, Heersink School of Medicine, University of Alabama at Birmingham, Birmingham, AL, United States; ^2^Department of Epidemiology, School of Public Health, University of Alabama at Birmingham, Birmingham, AL, United States; ^3^Department of Human Studies, School of Education, University of Alabama at Birmingham, Birmingham, AL, United States

**Keywords:** motor vehicle, dashboard, design, vision, visual function, older driver

## Abstract

**Purpose:**

Proper understanding and interaction with the dashboard is an essential aspect of safely operating a motor vehicle. A portion of this task is dependent on vision, yet no published information exists regarding dashboard ergonomics and visual function. This study sought to associate visual functions and person abilities of dashboard ergonomic dimensions relevant to older driver design preferences and attitudes.

**Methods:**

In this population-based study of drivers, participants completed functional testing for habitual distance visual acuity, contrast sensitivity, visual field sensitivity, visual processing speed, and spatial ability. A questionnaire assessed attitudes and understanding of dashboard design, with questionnaire items generated from the content of focus groups of older drivers. Dashboard design domains identified in Rasch analysis of questionnaire responses were quantified using person ability measures for the cognitive processing, lighting, obstructions, and pattern recognition domains. Visual functions and person abilities were correlated using Spearman partial correlations, adjusting for age and sex.

**Results:**

A total of 997 participants completed functional testing and the dashboard questionnaire. The mean age was 77.4 ± 4.6 years, and the majority were male (55%) and white (81%). The sample had a range of person abilities and visual functions. Contrast and visual field sensitivities were significantly associated with the cognitive processing, lighting, and pattern recognition dashboard design dimensions (*p* ≤ 0.0052). For all significant associations, increased visual function was indicative of better person ability. Visual processing speed, as measured by Trails B and UFOV2, was significantly associated with the lighting domain (*p* = 0.0008 and *p* = 0.0007, respectively). The UFOV2 measure was correlated with pattern recognition (*p* = 0.0165). Spatial ability was the only visual function associated with the visual obstruction dimension (*p* = 0.0347).

**Conclusions:**

Person ability for dashboard design domains are related to visual function in older drivers. Results show person ability for domains increased with improved visual function. Future automotive engineering and design initiatives should consider these associations in improving dashboard designs to increase vehicle utility and accessibility.

## Introduction

Instrument clusters in motor vehicles (known as the “dashboard”) are a primary interface for human and vehicle interaction during driving and are essential for safe operation of the vehicle. Early in the design of dashboards, they were simplistic, relaying limited information to the driver. But advancements in technology and demands from consumers have greatly expanded what is displayed and controlled from the console (Hind, 2021). The interaction of the driver and dashboard is critically dependent on vision, yet there has been little to no work on how the driver's visual functional abilities impact their perception and understanding of the dashboard. This is particularly true for older drivers, who have high prevalence of vision impairment compared to younger populations (Rubin et al., 1997; Haegerstrom-Portnoy et al., 1999).

Some research has focused on the visual demand of the dashboard, which is a measure of visual workload and not visual function *per se* (Strayer et al., 2019a,b). For example, time was measured for drivers to complete a detection response task, such as responding to a changing light or vibration presented every 3–5 s, while simultaneously driving and completing an in-vehicle information system task, like adjusting audio entertainment (Strayer et al., 2019b). This estimated the additional demand needed for the in-vehicle information system task. Most human factor research and guidelines focus on specifications for lettering or symbols like size, spacing, contrast, and luminance (National Highway Traffic Safety Administration and DOT, 2003; Campbell et al., 2004). These specifications relate to visual acuity and contrast sensitivity, two pertinent measures of foveal vision. Although a few research studies have evaluated participants for visual acuity (Steinfeld et al., 1996; Dobres et al., 2016), these assessments have the purpose of excluding drivers with acuity impairment from the study sample. This work ignores the research question of how common vision impairments among older drivers impact their attitudes about dashboard design.

Previous work has shown that older drivers with vision impairment are at increased risk of motor vehicle collisions (Owsley et al., 1998; Friedman et al., 2013; Huisingh et al., 2014; Swain et al., 2021b). Worse on-road performance is also associated with poorer vision in older adults (Swain et al., 2021a). Although vision is associated with both driver safety and performance in older drivers, no published work has examined the associations between vision and dashboard design in this age group. The purpose is to understand how dashboard design could be enhanced to meet the visual characteristics of this population. With rapid advancements in motor vehicle technology and design, the opportunity exists for design to be reflective and inclusive of more drivers, particularly older drivers who represent a large segment of the driving population. As vehicles transition to digital displays and controls, there are many opportunities for enhancements in the dashboard's configurable displays, and importantly, designs can be considered that are driver-specific customizations. With little information available on how vision is related to drivers' perceptions and understanding of dashboards, this older driver study is aimed to assess the associations between various types of visual function and attitudes, or ways of thinking or feeling, about perceiving and understanding dashboard design. We hypothesize that better visual function is associated with increased person ability for dashboard domains.

## Methods

### Study design and population

Detailed information of this prospective cohort study has been previously published (Owsley and McGwin, 2013). Briefly, potential participants ≥70 years old in Jefferson County, Alabama and surrounding counties were identified at random. A random selection of those with a valid driver's license from the State of Alabama were contacted *via* mail and later telephoned to confirm meeting study criteria. The sample had 2,000 enrollees, however only the last 1,000 enrollees were administered the Dashboard Questionnaire and comprised the sample for this analysis. The study was approved by the Institutional Review Board of the University of Alabama at Birmingham and adhered to Declaration of Helsinki.

### Participant assessment

At baseline, interviewer-administered questionnaires were conducted. Participants provided demographic and education information, and a questionnaire assessed general health for 17 common health conditions (e.g., diabetes, cancer, heart disease; Owsley et al., 2002). Testing for habitual distance visual acuity, contrast sensitivity, visual field sensitivity, visual processing speed, and spatial ability was also completed. Unless otherwise noted, all vision tests were done binocularly. Habitual distance visual acuity was assessed using the Electronic Visual Acuity system (JAEB Center for Health Research, Tampa, FL) and expressed as the log of the minimum angle of resolution (logMAR; Beck et al., 2003). Visual acuity impairment was defined as logMAR worse than 0.30 (20/40), the licensing standard used for driving by many government jurisdictions. The Pelli-Robson chart was used to assess contrast sensitivity under photopic conditions, was scored using the letter-by-letter method, and expressed as log sensitivity (Pelli et al., 1988; Elliott et al., 1991). Contrast sensitivity values <1.5 log sensitivity were considered impaired (Elliott et al., 1991). The Useful Field of View subtest 2 (UFOV2; Visual Awareness Research Group, Punta Gorda, FL) evaluated visual processing speed under divided attention (Edwards et al., 2006). In this test, the time in milliseconds (ms) required for the participant to discriminate between two targets in central vision while concurrently identifying the location of a peripheral target at a 10-degree eccentricity at one of eight potential radial locations is measured. UFOV2 times 150–350 ms were considered moderately impaired and those >350 ms severely impaired (Friedman et al., 2013). The Trails *B* test evaluated visual processing speed under divided attention in conjunction with executive function and working memory (Reitan, 1955). This test measures the time in minutes for a participant to draw a line from numbers and letters alternately while following numerical and alphabetical orders. Trails B times ≥2.47 min were impaired (Goode et al., 1998). Visual field sensitivity was assessed using a custom test on the Humphrey Field Analyzer, Model II-I (Carl Zeiss Meditec, Dublin, CA; Huisingh et al., 2014), and was assessed for each eye separately. This custom test assessed 20 locations with target locations chosen to be representative of the visual field while driving (Vargas-Martín and García-Pérez, 2005; Huisingh et al., 2014). Targets were located up to 60° horizontally, 15° superiorly, and 30° inferiorly. Monocular fields were combined to form a binocular visual field, which consisted of 21 test targets located up to 60° horizontally, 15° superiorly, and 30° inferiorly. Binocular visual field sensitivity at each location was defined by the most sensitive point of the two eyes. For the locations at 60° temporally on the horizontal meridian, the sensitivity was defined by the respective eye. The visual field measure in this analysis, expressed in decibels (dB), was the mean of all binocular visual field sensitivities. Lastly, spatial ability was evaluated using the Visual Closure Subtest of the Motor-Free Visual Perception Test (MVPT) and expressed as number scored correctly out of 11 (Colarusso and Hammill, 2003). This test examines one's ability to recognize incompletely drawn objects by matching them to completely drawn versions of the object. MVPT scores <8 were categorized as impaired (Ball et al., 2006).

The Dashboard Questionnaire (Swain et al., 2022) was administered *via* interview to assess participants' attitudes about aspects of dashboard design. In previous work, topics for the questionnaire were selected based on input from vehicle and human factors engineer experts in the design of vehicle instrument format clusters, scientists with expertise in vision, cognition, and aging, and occupational therapists specializing in driver rehabilitation (Owsley et al., 2011). Eight focus groups were held with older drivers. A semi-structured “script” (topics selected for discussion) was used by a trained facilitator to guide discussion, and a content analysis generated the content of specific items. Included topics in the questionnaire were control devices, instruments that gave the driver feedback about status of various vehicle functions, uniformity, interior lighting, font/lettering, color, word/symbols, location, entertainment and navigation. The response options used a Likert-scale of “Definitely True,” “Mostly True,” “Neutral,” “Mostly False,” and “Definitely False.” Responses to the questionnaire were evaluated using Rasch analysis (Rasch, 1960, 1980) to gain person ability measures about older drivers' attitudes about dashboard design (Swain et al., 2022). This analysis revealed that questionnaire item responses fell into four distinct domains: cognitive processing, lighting, visual obstruction, and pattern recognition. The person ability measures provided information about whether participants experienced problems (i.e., negative attitudes) with any of domains. Person ability measures, expressed on a logit scale, where lower scores indicate better ability and higher scores worse ability for the perceived construct, were derived for each domain and then associated with visual function. Table 1 lists the questionnaire items falling into each of the four dashboard domains.

**Table 1:** Dashboard questionnaire items evaluated and corresponding domain derived from Rasch analysis.

**Questionnaire item**	**Rasch domain**
Some symbols on my dashboard are difficult to understand.	Cognitive processing
Some abbreviations on knobs and switches are difficult to understand.	Cognitive processing
The dashboard is cluttered with too much information I don't use.	Cognitive processing
The dashboard is so cluttered with gauges, buttons, and controls that it is overwhelming.	Cognitive processing
I understand the meaning of the information presented by all my car's gauges.	Cognitive processing
Multi-purpose knobs and levers are confusing.	Cognitive processing
When I glance down at the dashboard, I have trouble finding what I'm looking for.	Cognitive processing
There are too many control knobs on the entertainment center (radio/CD/tape player).	Cognitive processing
The display on the entertainment center (radio/CD/tape player) is difficult to read.	Cognitive processing
The lettering is too small on the knobs, controls and switches.	Cognitive processing
Reading things on the dashboard takes my eyes off the road for too long.	Cognitive processing
I limit my driving at night because the dashboard lights cause a glare on the windshield.	Lighting
The blinker light (turn signal indicator) is too dim.	Lighting
The blinker light (turn signal indicator) is too small.	Lighting
When I'm driving at night, the lights illuminating the dashboard are too dim.	Lighting
I limit my driving at night because I have trouble seeing the dashboard.	Lighting
The gas gauge is too small.	Lighting
Knobs on the dashboard are too small.	Lighting
The steering wheel blocks my view of the blinkers (turn signal indicators).	Lighting
When I glance down at the dashboard, the gauges are blurry.	Lighting
When I glance down at the dashboard, the labels, symbols or letters are blurry.	Pattern recognition
The knobs on the dashboard are hard to see because they blend in with the background.	Pattern recognition
The labels, symbols, or letters on the dashboard need to be bigger.	Pattern recognition
Some controls (for example, buttons, knobs) are not within my reach while I'm driving.	Pattern recognition
The steering wheel blocks my view of the speedometer.	Obstruction
I have difficulty seeing my speedometer.	Obstruction
I have difficulty seeing my gas gauge.	Obstruction

### Statistical analysis

Participant demographic and visual functions were summarized using means and standard deviations or number and percent for continuous and categorical data, respectively. Cross-sectional analysis of baseline visual functions and Rasch person ability measures of dashboard questionnaire domains was completed using Spearman correlations, expressed as Spearman correlation coefficients (R_s_), adjusted for age and sex. The level of significance was set at *p* ≤ 0.05 (two-sided). All analyses were completed in SAS version 9.4 (SAS Institute, Cary, NC).

## Results

Of the 1,000 participants, 997 completed all visual function testing and were included for analysis. The majority of participants were aged 70–79 (70.5%) and male (55.2%; Table 2). Education level of participants varied with 31.1% not having completed high school, 2.8% with a high school degree or equivalent, 49.7% with some college or a college degree, and 10.8% completed processional or graduate school. The majority of participants had 2–3 medical conditions (44.5%) followed by 4–5 conditions (29.8%), 0–1 conditions (14.8%), and 6 or more (10.8%).

**Table 2:** Participant characteristics (*N* = 997).

	**Mean (standard deviation) or *N* (%)**
Mean age, years	77.4 (4.6)
Age group	
70–79	703 (70.5)
80–89	282 (28.3)
90–98	12 (1.2)
Sex	
Female	447 (44.8)
Male	550 (55.2)
Race	
White	805 (80.7)
Black	187 (18.8)
Other	5 (0.5)
Education category	
Less than high school	310 (31.1)
High school degree or equivalent	28 (2.8)
Some college or college degree	495 (49.7)
Professional or graduate school	108 (10.8)
Medical conditions, number	
0–1	148 (14.8)
2–3	444 (44.5)
4–5	297 (29.8)
≥6	108 (10.8)

Here we summarize each visual function. On average, all visual functions were normal; however, the sample had a range of function including those with impairment. The mean (standard deviation) visual acuity was 0.05 (0.14) logMAR (~20/25; Table 3). With logMAR worse than 0.3 (20/40) considered impaired, 8% of participants had acuity impairment. Contrast sensitivity had a mean of 1.67 (0.13) log sensitivity with values ranging from 0.75 to 1.95 log sensitivity. In the sample, 7.4% had impaired contrast sensitivity. The average processing speed under divided attention was 155.5 (133.5) ms as assessed by the UFOV subtest 2. UFOV2 times ranged from 17 to 500 ms, with 31% of participants moderately impaired and 9.6% severely impaired. Processing speed results evaluated from Trails B test were similar, with an average of 2.5 (1.4) min and 37% of the sample having worse times (≥2.47 min). Spatial ability had a mean score of 9.4 (1.6) out of 11. Spatial ability scores were worse, scores <8, in 13% of participants. Lastly, the average visual field sensitivity was 24.6 (3.2) dB with a range of 5.8–38.7 dB. Of note, for visual acuity, Trails B, and UFOV2, lower values are indicative of better function. Conversely, higher values of contrast sensitivity, visual field sensitivity, and spatial ability represent better function.

**Table 3:** Visual function and Rasch person abilities for dashboard domains of participants.

	**Mean (standard deviation)**
**Visual function**
Visual acuity, logMAR	0.05 (0.14)
Contrast sensitivity, log sensitivity	1.67 (0.13)
UFOV subtest 2, milliseconds	155.5 (133.5)
Trails B, minutes	2.5 (1.4)
MVPT, number correct score	9.4 (1.6)
Visual field sensitivity, decibels	24.6 (3.2)
**Person abilities for dashboard domains**	
Cognitive processing	−1.2 (1.6)
Lighting	−2.6 (2.0)
Obstruction	−2.8 (2.1)
Pattern recognition	−1.5 (1.7)

The mean Rasch person ability measures for the four dashboard ergonomics domains indicate mean abilities were fair to good with values ranging −1.2 to −2.8 logits (Table 3). The mean (standard deviation) person ability for the cognitive processing domain was −1.2 (1.6) with a range of abilities in the sample (−5.4 to 5.2). The pattern recognition domain had a mean of −1.5 (1.7) and values ranging −4.6 to 2.9. Person abilities, on average, were lower for the lighting and obstruction domains (means of −2.6 and −2.8, respectively) as compared to the other two domains, indicating participants found these domains less problematic. These domains also had a range of person abilities (lighting: −5.7 to 4.7; obstruction: −4.9 to 4.2).

Visual acuity was not associated with person ability for any dashboard questionnaire domains (Table 4). For all significant associations in Table 4, better visual function was associated with increased person ability in dashboard responses. The cognitive processing domain was associated with contrast sensitivity and visual field sensitivity (R_s_ = −0.10, *p* = 0.0016 and R_s_ = −0.09, *p* = 0.0064, respectively). The pattern recognition domain was also associated with contrast sensitivity and visual field sensitivity (R_s_ = −0.09, *p* = 0.0052 and R_s_ = −0.08, *p* = 0.0165, respectively), and also UFOV2 (R_s_ = 0.07, *p* = 0.0219). The lighting domain was significantly correlated with all visual functions (except for acuity), with correlations ranging from 0.09 to 0.11 and *p* ≤ 0.0064. Spatial ability was the only functional measure associated with the visual obstruction (R_s_ = −0.09, *p* = 0.0037).

**Table 4:** Associations of person abilities of dashboard ergonomic domains with visual functions, adjusted for age and gender.

	**Cognitive processing**		**Lighting**		**Obstruction**		**Pattern recognition**	
	**R_s_**	***p*-value**	**R_s_**	***p*-value**	**R_s_**	***p*-value**	**R_s_**	***p*-value**
Visual acuity	0.01	0.8588	0.02	0.5149	0.03	0.2739	0.05	0.1529
Contrast sensitivity	−0.10	0.0016	−0.10	0.0023	−0.05	0.1177	−0.09	0.0052
Visual field sensitivity	−0.09	0.0064	−0.09	0.0064	−0.05	0.1054	−0.08	0.0165
Trails B	0.03	0.3451	0.11	0.0008	0.05	0.0896	0.04	0.2646
MVPT	−0.05	0.1516	−0.09	0.0037	−0.07	0.0347	−0.05	0.0973
UFOV2	0.03	0.3490	0.11	0.0007	0.06	0.0690	0.07	0.0219

*R_s_, Spearman correlation coefficient*.

## Discussion

Worse visual function in a population-based sample of drivers aged 70 and older is associated with lower person abilities in their attitudes about dashboard design in vehicles. This implies that the integrity of visual function is important for understanding how older drivers perceive and understand the dashboard. All visual functions were associated with person ability scores in various domains, except for visual acuity. One may speculate that spatial resolution (acuity) may not have been a limiting factor for older drivers in interacting with dashboards, especially since their visual acuity deficits that occurred were not severe.

Visual obstruction on the dashboard, i.e., near objects visually obstructing objects further away, was related to spatial ability. Those with worse spatial ability scores were more likely to report problems with visual obstructions. When viewing a visually obstructed object, the perceiver is asked to understand the distal, rather than the retinal, spatial arrangements of objects. This is the visuo-cognitive skill that the visual closure subtest of the MVPT actually addresses (Colarusso and Hammill, 2003), so it is interesting that this skill is associated with perceiving and understanding visual obstructions on the dashboard. No other measures of visual function were associated with the visual obstruction domain.

Expressing problems in cognitive processing domain items was associated with decreased contrast sensitivity and visual field sensitivity. Cognitive processing relies heavily on obtaining reliable information about the world, with two key visual skills being contrast sensitivity (the building block of vision; Owsley, 2003; Pelli and Bex, 2013) and understanding the visual world throughout the visual field. Similarly, achieving reliable and valid visual pattern recognition relies on contrast sensitivity and apprehension of the visual field, and thus it is not surprising that these two visual skills were also associated with the pattern recognition domain. All visual functions (except for visual acuity) were related to person ability scores in the lighting domain. Lighting impacts all aspects of visual function since the relative contributions of cone vs. rod photoreceptor function to vision is driven by the background lighting level of the environment.

Our work suggests older drivers are an important demographic for guiding dashboard development given visual declines are related to their person abilities in processing dashboard information. This also recognizes the concept of universal design in that dashboard designs that facilitate older drivers abilities to process dashboard information can be appropriate for all drivers regardless of visual status (Campbell et al., 2004; Herriotts, 2005; Dickerson et al., 2007). Older adults as a group typically have much longer driving histories as compared to younger drivers (Young et al., 2017). Yet, older adults often have greater challenges in adapting to new technologies than younger adults (Dickerson et al., 2007). Older adults are at higher risk, as compared to younger adults, for problems in coping with visual distractions and making decisions under conditions of uncertainty (Yang and Coughlin, 2014). Despite slower acceptance of new technological systems, ironically, older adults are more likely to have newer vehicles, due to increased financial security (Young et al., 2017). Given these characteristics, older drivers may be valuable for evaluating technologically novel dashboard designs.

It is not immediately obvious that automotive research developing regulations and guidelines for dashboard design are based on well-powered samples, variable participant visual function, and testing of visual function other than acuity. It has been suggested that dashboard design has been by consensus, which is a questionable method (Campbell et al., 2004). With some symbols used today having been tested on a convenience sample of car manufacturing employees (Frank et al., 1973), even those who were not drivers, and among plant visitors, evaluations have lacked rigor. Though proprietary concerns may prevent publication of current design processes, it would only behoove automobile makers to ensure designs are inclusive of more disparate groups of drivers. While our work focused on drivers 70 and older, the findings presented are relevant to many drivers since the prevalence of vision impairment is increasing, with 8 million people projected to have visual impairment or blindness in the U.S. by 2050 (Varma et al., 2016). As dashboards evolve to more advanced display systems, the opportunity exists to improve the utility of future vehicles with well-tested dashboard designs.

Strengths of our study are its population-based nature and its large sample. No previous studies have examined the role of vision problems in older drivers' reactions to key characteristics of vehicle dashboard design, with the ultimate goal of providing guidance to vehicle dashboard designers so they can be more attuned to the visual characteristics of drivers. We utilized a Rasch analysis since it is used to measure latent traits like attitudes or ability; it allows research to use a respondent's raw test score and express the respondent's performance on a linear scale that accounts for unequal difficulties across all test items. A limitation of our study is that the associations between visual function and dashboard design domains, although statistically significant, were low. Many factors influence drivers' attitudes about dashboard design, such as personal history with their own personal vehicle and their experience with other vehicle types, the extent to which they utilize various features of the dashboard, and their preferences for simplicity vs. complexity of dashboard design. However, we believe we have clearly established that the visual characteristics of older drivers do influence their attitudes and beliefs about dashboard design. Lastly, cognitive function was not considered in this analysis.

In conclusion, person ability in the cognitive processing, lighting, obstruction, and pattern recognition domains in dashboard design are significantly associated with several measures of visual function. Automotive manufacturers may find it useful to develop and evaluate dashboard designs by including older drivers, since vision impairment is more common in this subpopulation, though research should involve any aged driver with visual impairment. More careful consideration and evaluation of vision in design may increase vehicle utility, safety, and satisfaction for all drivers.

## Data availability statement

The raw data supporting the conclusions of this article will be made available by the authors, without undue reservation.

## Ethics statement

The studies involving human participants were reviewed and approved by Institutional Review Board of the University of Alabama at Birmingham. The participants provided their written informed consent to participate in this study.

## Author contributions

GM and CO conceptualized and administrated the overall study. TS, GM, and CO contributed to the design and conceptualization of the topic. SS completed the Rasch analysis and generated the person ability measures. TS completed the statistical analysis with oversight by GM. TS and CO drafted the manuscript. All authors revised the manuscript and approved the final version.

## Funding

This work was supported by National Institutes of Health R01EY018966, P30AG022838, and P30EY003039, General Motors (PRVA0258), EyeSight Foundation of Alabama, and Research to Prevent Blindness.

## Conflict of interest

The authors declare that the research was conducted in the absence of any commercial or financial relationships that could be construed as a potential conflict of interest.

## Publisher's note

All claims expressed in this article are solely those of the authors and do not necessarily represent those of their affiliated organizations, or those of the publisher, the editors and the reviewers. Any product that may be evaluated in this article, or claim that may be made by its manufacturer, is not guaranteed or endorsed by the publisher.
